# Changes in the renin angiotensin system during the development of colorectal cancer liver metastases

**DOI:** 10.1186/1471-2407-10-134

**Published:** 2010-04-10

**Authors:** Jaclyn H Neo, Eleanor I Ager, Peter W Angus, Jin Zhu, Chandana B Herath, Christopher Christophi

**Affiliations:** 1 Department of Surgery, The University of Melbourne, Austin Health, Heidelberg, Victoria, Australia; 2 Department of Medicine, The University of Melbourne, Austin Health, Heidelberg, Victoria, Australia

## Abstract

**Background:**

Blockade of the renin angiotensin system (RAS) via angiotensin I converting enzyme (ACE) inhibition reduces growth of colorectal cancer (CRC) liver metastases in a mouse model. In this work we defined the expression of the various components of the RAS in both tumor and liver during the progression of this disease.

**Methods:**

Immunohistochemistry and quantitative RT-PCR was used to examine RAS expression in a mouse CRC liver metastases model. CRC metastases and liver tissue was assessed separately at key stages of CRC liver metastases development in untreated (control) mice and in mice treated with the ACE inhibitor captopril (750 mg/kg/day). Non-tumor induced (sham) mice indicated the effect of tumors on normal liver RAS. The statistical significance of multiple comparisons was determined using one-way analysis of variance followed by Bonferroni adjustment with SAS/STAT software.

**Results:**

Reduced volume of CRC liver metastases with captopril treatment was evident. Local RAS of CRC metastases differed from the surrounding liver, with lower angiotensin II type 1 receptor (AT1R) expression but increased ANG-(1-7) receptor (MasR) compared to the liver. The AT1R localised to cancer and stromal infiltrating cells, while other RAS receptors were detected in cancer cells only. Tumor induction led to an initial increase in AT1R and ACE expression while captopril treatment significantly increased ACE expression in the final stages of tumor growth. Conversely, captopril treatment decreased expression of AT1R and angiotensinogen.

**Conclusions:**

These results demonstrate significant changes in RAS expression in the tumor-bearing captopril treated liver and in CRC metastases. The data suggests the existence of a tumor-specific RAS that can be independently targeted by RAS blockade.

## Background

Colorectal cancer (CRC) is a leading cause of cancer death worldwide, with approximately 940 000 new cases and 500 000 deaths reported annually [1]. Mortality from CRC is primarily due to metastasis to the liver, accounting for over 70% of deaths [2]. Surgical resection provides the best chance of cure. However, only 20% to 25% of patients are eligible for surgery, with recurrence rates approaching 40% to 70% [3,4]. Palliative systemic chemotherapy is the preferred option for the majority of these patients.

Recent studies suggest that therapies targeting paracrine hormone systems that promote tumor development may provide an alternative or additional treatment strategy in these patients. There is strong evidence that long term blockade of the renin angiotensin system (RAS) in hypertensive patients is associated with a decreased incidence of several human cancers [5]. Evidence also suggests that blockade of the RAS in experimental animal models of CRC liver metastases is associated with tumor growth inhibition [6-9]. The angiotensin I converting enzyme (ACE) is a key enzyme in the RAS, cleaving the biologically inactive angiotensin (ANG) I precursor to ANG II, the key effector peptide of the RAS. ACE inhibition is associated with a reduction in tumor growth for several malignancies including breast, prostate, lung, and colon cancer [5,10-12].

In the liver, the local RAS is up-regulated in response to tissue injury and hypoxia [13,14]. However, its expression during the development of CRC liver metastases has not been examined. ANG II stimulates the expression of several growth and pro-angiogenic factors including vascular endothelial growth factor (VEGF) [15,16]. The pro-angiogenic effects of ANG II are mediated by the ANG II type 1 receptor (AT1R), which is overexpressed in several human cancers [7-19].

Recent studies demonstrate that while ANG II/AT1R signalling has proliferative and angiogenic effects, counter-regulatory effects are mediated by other RAS components. For example, activation of the AT2R, which is expressed in preference to AT1R in primary CRC, inhibits angiogenesis and cellular proliferation [20,21]. In addition, a homologue of ACE, ACE2, was recently described and its expression is up-regulated in liver injury [13,22-24]. This enzyme generates the peptide ANG-(1-7) directly from ANG II and indirectly from ANG I [23]. ANG-(1-7) acts through the MasR (*m*itochondrial *as*sembly receptor) and appears to antagonize some ANG II-induced effects, including angiogenesis and cellular proliferation [25-27]. Although ANG-(1-7) decreases proliferation of several cell types, including human lung cancer cells *in vitro *[28], it has also been associated with increased cellular proliferation [29,30].

Several components of the RAS are expressed in primary CRC and we have shown previously that blockade of the RAS decreased tumor growth in a mouse model of CRC liver metastases [8,19]. However, the ontogeny of RAS expression during CRC liver metastases progression has not been described, nor have the effects of captopril treatment on RAS expression in tumors been documented. This study aimed to establish an expression profile of the RAS in both captopril treated and untreated early, mid, and late stages of CRC liver metastases.

## Methods

### Animals

Male inbred 6-8 week old CBA mice were obtained from Adelaide University Animal Facility, Australia. Experiments were performed according to the Austin Health Animal Ethics Committee guidelines.

### Mouse model of colorectal liver metastases

A mouse model of CRC liver metastases was used as described previously [31]. A dimethyl-hydrazine-induced primary colon carcinoma was maintained by *in vivo *serial passage in the flanks of male CBA mice. Tumors were removed from passage mice and used to make a tumor cell suspension (1 × 10^6 ^cells/ml in Ringers solution/0.1% glucose). For tumor-induction, mice were anaesthetized with an intraperitoneal injection (0.1 ml/10 mg body weight) of ketamine/xylazine. The spleen was exteriorized through a subcostal incision. Tumor cell suspension (0.05 ml) was slowly injected into the spleen using a 25-gauge needle over a period of 1 minute. The needle was retracted and even pressure was applied to the spleen for 2 minutes. A haemostatic clip sealed the splenic vessels and a splenectomy was performed. The skin was sutured and the mouse recovered on a heated pad. Sham mice had a 0.05 ml injection of Ringers/0.1% glucose solution and a splenectomy.

This model has been characterized previously and results in metastases exclusively confined to the liver [31]. Angiogenesis is established by day 10, followed by exponential growth of tumors between days 10-16, and a plateau phase from day 19-22.

### Sample collection

Liver samples from 3-5 animals per time point and treatment were collected at 5, 10, 16 and 21 days after CRC liver metastases induction. However, because of the small size of tumors in the early stages (days 5 and 10), analysis of CRC metastases was restricted to days 16 and 21. All effort was made to completely separate tumor from adjacent liver; however, contamination from micro metastases cannot be excluded from samples of the tumor-bearing liver.

### Captopril treatment

Captopril (D-3-mercapto-2-methylpropanoyl-L-proline, Sigma-Aldrich, Sydney, NSW, Australia) was made fresh in PBS. The dosing regime was as described previously [8]. Briefly, captopril was administered daily to mice via intraperitoneal injection (750 mg/kg; at a volume of 0.3 ml). Dosing began on the day of CRC liver metastases induction and continued daily until the experimental endpoint. Control mice received an equivalent volume of PBS.

### RNA extraction and cDNA synthesis

Liver and CRC metastases tissues were homogenized in 1 ml of Trizol (Invitrogen) to obtain total RNA. RNA was DNase treated (Ambion, DNA-free™) to remove contaminating genomic DNA. RNA concentration and purity were determined using a spectrophotometer. Approximately 1 μg of total RNA, 100 ng of random hexamers (Invitrogen), 2 μl deoxyribonucleotide triphosphate (10 mM dNTP), and 2000 U of reverse transcriptase were used in a cDNA synthesis reaction.

### Quantitative RT-PCR (qRT-PCR) analysis

An aliquot of cDNA (between 5 and 100 ng of cDNA according to primer efficiency and the anticipated level of expression for each gene) was used in a qRT-PCR (ABI Prism 7700 Sequence Detector). To confirm the absence of contaminating genomic DNA, RT-minus and no template controls were performed. Probes and primers for mouse tissue were designed using Primer Express, Ver 1.0 (PE Applied Biosystems, Foster City, CA) and ordered from Geneworks (Table 1). Each cDNA sample was analyzed in duplicate and assessed using the comparative C_T _method. The qRT-PCR protocol began with a 50°C incubation for 2 minutes, followed by 95°C for 10 minutes, and 45 cycles at 95°C for 15 seconds and 60°C for 1 minute. 18S ribosomal RNA served as the internal control. Values from non-treated and treated liver and liver metastases were normalized against sham livers, which were given a value of 1.

**Table 1:** Primer and probe sequences for qRT-PCR listed from the 5' to 3' direction.

Gene	Primer	Size (bp)	Probe	Size (bp)
Mas R	Forward TGTGGGCACTTTCGTGCTT Reverse AATGACTCTCTTCTCCGCTGTCA	19 23	CACCATGGAGTATGTCATGT	20
ACE	Forward CAGAATCTACTCCACTGGCAAGGT Reverse TCGTGAGGAAGCCAGGATGT	24 20	CAACAAGACTGCCACCTGCTGGTCC	25
ACE2	Forward TGCCCATTTGCTTGGTGAT Reverse AAAGGGAACAGTCAAAGGGTACAG	19 24	TTTGTCCAAAATCTACCCCACA	22
AT1R	Forward GGGCAGTTTATACCGCTATGGA Reverse TGGCCGAAGCGATCTTACAT	22 20	TACCAGTGGCCCTTCGGCAATCA	23
AT2R	Forward ATTACCTGCATGAGTGTCGATAGG Reverse AGATGCTTGCCAGGGATTCC	24 20	ACCAATCGGTCATCTACCCTTTTCTGTCTC	30
Angioten-siongen	Forward CTGCTCCAGGCTTTCGTCTAA Reverse AGAACTGGGTCAGTGGATAAATCC	21 24	CCCTGCCCTCTTCCCACGCTCTC	23

### Immunohistochemistry

To measure RAS proteins, samples were collected in 4% paraformaldehyde and fixed for 48 hours before embedding in paraffin. Sections were cut at 4 μm and mounted on SuperFrost Plus slides (Menzel-Glaser). Sections were dewaxed in two 10 minutes histolene solvent baths and rehydrated by serial progression through three 100% EtOH baths. Endogenous peroxidase activity was blocked with 3% hydrogen peroxide in TBST for 5 minutes. For the ACE H-170 antibody (rabbit polyclonal, Santa Cruz, cat# sc-20791) and the AT2R antibody (rabbit polyclonal, Santa Cruz, cat# sc-9040), an antigen retrieval step in citrate buffer (10 mM, pH 6) for 15 and 10 minutes, respectively, at low microwave power was required. AT1R and MasR immunostaining did not require antigen retrieval. The ACE H-170 antibody was applied at 0.001 g/L, the AT1R antibody (rabbit polyclonal, Santa Cruz, cat# sc-1173) at 1 μg/mL, the AT2R antibody at 2.5 μg.mL, and the MasR antibody (rabbit polyconal, Lifespan Biosciences, cat# LS-A1528) at 10 μg/mL. Control sections were incubated in diluent or appropriate IgG negative control. Slides were incubated at 37°C for 2 hours (ACE, AT1R and the MasR antibody) or for an hour (AT2R antibody) followed by an overnight 4°C incubation. Slides were then incubated for 1 hour at 37°C with an anti-rabbit horseradish peroxidase secondary antibody (Dako, cat# 4011). The final detection step was carried out using 3,3'-diaminobenzidine (DAB, Dako, cat# 4011) as the chromogen. Sections were counterstained with Mayer's hematoxylin for 3 minutes and cover slipped.

### ACE positive cell counts

The Nikon Coolscope digital microscope and Image Pro Plus image analysis system were used to quantify the number of cells in the tumor stroma and used each nucleus that was associated with surrounding ACE positive staining to distinguish individual cells in treated and un-treated tumors. Positively staining cells were counted per high-powered field (20 × magnification) and measured relative to tumor area. For each section, between 7 and 40 images were captured (depending on the number/size of tumors). Cell counts were averaged to give a single value for each section/individual. Between 4 and 6 individuals were assessed at each stage. Cell counts were performed on day 16 and 21 tumors, but not in the adjacent liver as ACE positive staining localized to non-sinusoidal endothelial cells in all treatments and conditions and differences in staining intensity could not be assessed with the antibody used.

### Statistical analyses

The statistical significance of multiple comparisons was determined using one-way analysis of variance followed by Bonferroni adjustment with SAS/STAT software. Quantitative data are presented as means ± s.e.m.. A *P*-value less than 0.05 was defined as significant.

## Results

### Captopril inhibits CRC liver metastases

**Figure 1:**
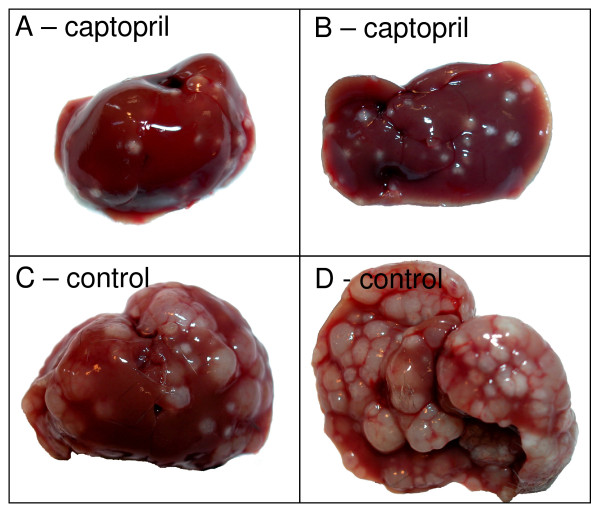
**An example of CRC liver metastases inhibition by captopril treatment**. Captopril treated livers (e.g. A, dorsal; B, ventral) had noticeably fewer metastases than the corresponding tumor-bearing controls (e.g. C, dorsal; D, ventral). Control and captopril images are to the same scale. These results confirm our previous study which described in detail the inhibitory effects of captopril on CRC liver metastases [8].

We previously described in detail the inhibitory effect of pharmacological blockade of the RAS on CRC liver metastases. We found that captopril administered daily at a dose of 750 mg/kg dramatically decreased the number and volume of tumors in a mouse model of CRC liver metastases [8]. In the present study, we again administered captopril using the same dosing regimen and animal model. Our present results confirm the previously documented inhibitory effect of captopril on CRC liver metastases (an example from day 21 is shown; Figure 1).

### RAS expression in the liver and colorectal metastases

**Figure 2:**
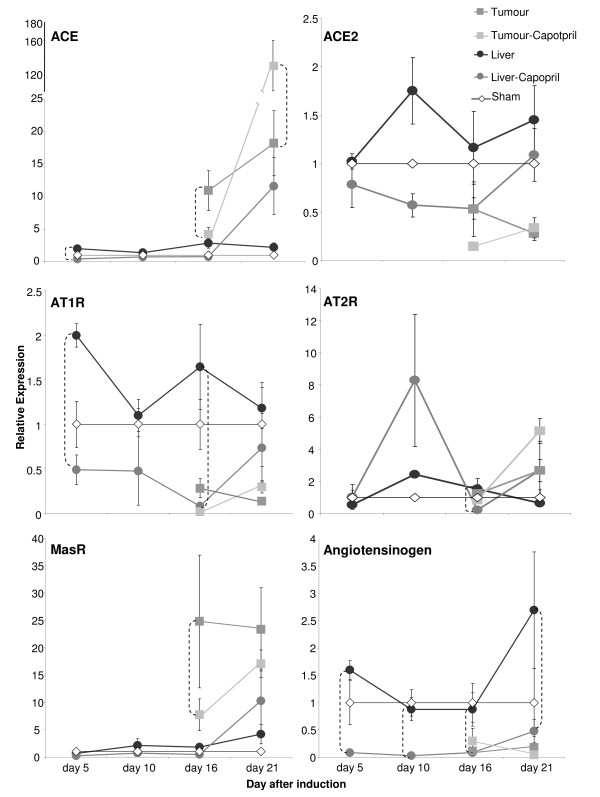
**Quantification of RAS mRNA expression by RT-PCR in sham livers, captopril-treated and control CRC liver metastases (tumors), and captopril-treated and control tumor-bearing liver (i.e. the liver surrounding tumors)**. Data for tumors is only presented at days 16 and 21 after induction as the small size of tumors was prohibitive for accurate analysis at earlier stages. Sham livers were standardized to 1 and other values normalized against sham for each time point. Significance between captopril treated and control animals is shown by connecting dotted vertical lines. The significance of other comparisons is presented in Table 2. Only ACE2 was not significantly altered either in CRC metastases or in the liver by captopril treatment.

While the liver adjacent to CRC metastases was assessed at days 5, 10, 16 and 21 by qRT-PCR, due to the small tumor size at days 5 and 10, CRC metastases samples were assessed only at days 16 and 21. Both CRC metastases and liver samples have been normalized against the same sham livers at each time point. Statistical significance for all comparisons is presented in Table 2, while Figure 2 indicates significant differences between control and treatment groups only.

**Table 2:** Bonferroni adjusted t-tests for comparisons between RAS gene expression in tumors, tumor-induced liver, and sham livers either with or without captopril treatment.

Gene	Comparison	Day 5	Day 10	Day 16	Day 21
ACE	Tumor control cf. sham			**0.0003**	0.3693
	Tumor control cf. tumor captopril			**0.0033**	**0.0001**
	Tumor control cf. liver control			**0.0007**	0.3508
	Liver control cf. sham	**0.0412**	0.5001	0.4026	0.9496
	Liver control cf. liver captopril	**0.0005**	0.2495	0.2839	0.6212
	Liver captopril cf. sham	0.1202	0.5906	0.9216	0.6110
	Tumor captopril cf. sham			0.1482	**0.0001**
	Tumor captopril cf liver captopril			0.0796	**0.0001**
ACE2	Tumor control cf. sham			0.2276	0.0531
	Tumor control cf. tumor captopril			0.2609	0.8660
	Tumor control cf. liver control			0.9828	**0.0011**
	Liver control cf. sham	0.9305	0.4077	0.6531	0.2105
	Liver control cf. liver captopril	0.3797	0.2270	0.0598	0.3130
	Liver captopril cf. sham	0.4554	0.6545	0.2146	0.8171
	Tumor captopril cf. sham			**0.0294**	0.0838
	Tumor captopril cf liver captopril			0.2259	0.0514
Ang	Tumor control cf. sham			**0.0119**	0.3345
	Tumor control cf. tumor captopril			0.7069	0.8632
	Tumor control cf. liver control			**0.0134**	**0.0024**
	Liver control cf. sham	0.0923	0.6725	0.6830	0.0530
	Liver control cf. liver captopril	**0.0004**	**0.0120**	**0.0108**	**0.0090**
	Liver captopril cf. sham	**0.0160**	**0.0052**	**0.0098**	0.5520
	Tumor captopril cf. sham			**0.0043**	0.2801
	Tumor captopril cf liver captopril			0.7858	0.5997
AT1R	Tumor control cf. sham			0.1071	**0.0223**
	Tumor control cf. tumor captopril			0.4844	0.6327
	Tumor control cf. liver control			**0.0019**	**0.0003**
	Liver control cf. sham	**0.0030**	0.8065	0.1292	0.6180
	Liver control cf. liver captopril	**0.0001**	0.1617	**0.0003**	0.2222
	Liver captopril cf. sham	0.0787	0.2352	**0.0360**	0.5013
	Tumor captopril cf. sham			**0.0261**	0.0677
	Tumor captopril cf liver captopril			0.8570	0.2443
AT2R	Tumor control cf. sham			0.7637	0.2765
	Tumor control cf. tumor captopril			0.4973	0.9972
	Tumor control cf. liver control			0.6145	0.0870
	Liver control cf. sham	0.4505	0.2186	0.4791	0.8018
	Liver control cf. liver captopril	0.4651	0.6104	**0.0434**	0.1241
	Liver captopril cf. sham	0.9716	0.4497	0.2960	0.2765
	Tumor captopril cf. sham			0.8127	**0.0086**
	Tumor captopril cf liver captopril			0.2897	0.0749
MasR	Tumor control cf. sham			**0.0090**	**0.0033**
	Tumor control cf. tumor captopril			**0.0299**	0.3405
	Tumor control cf. liver control			**0.0036**	**0.0046**
	Liver control cf. sham	0.4294	0.2097	0.9185	0.6467
	Liver control cf. liver captopril	0.2900	0.1486	0.8491	0.3546
	Liver captopril cf. sham	0.0902	0.7769	0.9501	0.2062
	Tumor captopril cf. sham			0.3995	**0.0339**
	Tumor captopril cf liver captopril			0.2990	0.3248

Tumor comparisons have only been made for days 16 and 21 due to the difficult in obtaining sufficient samples at earlier stages (days 5 and 10). Comparisons between liver tissues, however, have been made at all stages (Days 5, 10, 16 and 21 post-tumor induction). Values were considered significant for P < 0.05 (highlighted in bold).

Key components of the RAS, namely angiotensinogen, ACE, ACE2, AT1R, AT2R, and the MasR were expressed in the sham liver, tumor-induced liver, and in CRC liver metastases (tumors) at all stages examined. Standard deviations between replicate samples of the same cDNA were less than 1 cycle, demonstrating the accuracy of the qRT-PCR analyses performed. Variation between liver samples between individuals may reflect contamination from micrometastses or standard biological variation, while deviations in CRC metastases samples likely reflect the heterogeneous nature of these tumors.

### Angiotensinogen expression is lower in CRC liver metastases and is reduced in the liver surrounding tumors following captopril treatment

Angiotensinogen mRNA expression in the liver surrounding CRC metastases was not significantly different from sham, indicating no effect of tumor-induction on the expression of this gene. Angiotensinogen expression in CRC metastases was unchanged by captopril treatment, with no significant difference between control and captopril treated CRC metastases at either day 16 or day 21. However, in the liver surrounding CRC metastases treatment with captopril resulted in significantly lower angiotensinogen mRNA expression at almost all time points compared to both the sham and control tumor-induced liver (*P *≤ 0.0160) (Figure 2 and Table 2).

### ACE and ACE2 expression in CRC liver metastases

A significant increase in ACE mRNA expression was observed at day 5 in the tumor-bearing liver compared to sham livers (*P *= 0.0412), indicating an effect of tumor induction. However, the highest ACE expression levels were observed in CRC metastases. ACE mRNA expression was higher in CRC metastases compared to the surrounding liver at day 16 (*P *= 0.0007) and was increased markedly at day 21 by captopril treatment; captopril treated CRC metastases had significantly higher ACE expression compared control CRC metastases (*P *= 0.0001) (Figure 2) as well as to the surrounding liver (*P *= 0.0001).

**Figure 3:**
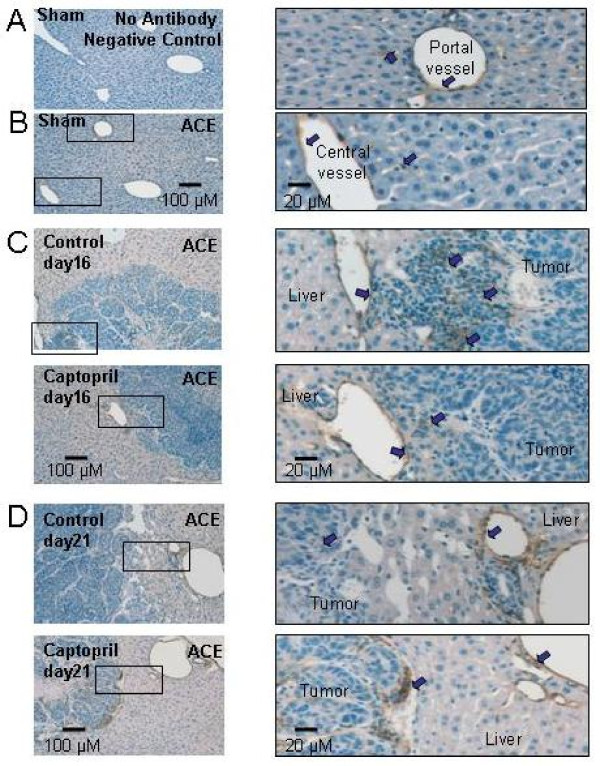
**Immunohistochemical staining against ACE in the sham (B), control and captopril-treated animals at day 16 (C) and 21 (D)**. No staining was evident in the negative control (A). The sinusoidal, portal vein, and central vein endothelium stained positively for ACE in the sham liver (B), and two images at higher magnification on the right). At day 16, there appeared to be more positive staining in control tumors compared to treated tumors (C). In contrast, at day 21 more cells stained positively for ACE when treated with captopril than when untreated (D).

**Figure 4:**
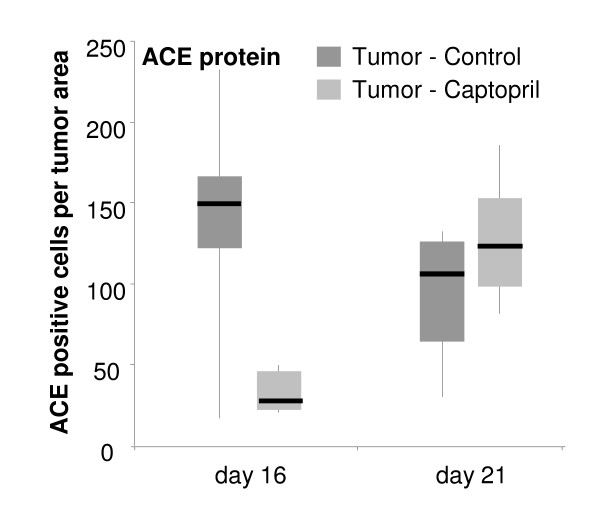
**ACE-positive cell counts in captopril treated and untreated tumors at days 16 and 21 after tumor-induction**. Because all vascular endothelial cells stained in the liver regardless of treatment with captopril, cell counts for the corresponding normal liver were not performed. Changes in the number of ACE positive cells in response to captopril treatment was similar to ACE mRNA (see Figure 2), with greater numbers and mRNA expression in untreated CRC metastases at day 16, but an increase in mRNA expression and number of ACE-positive cells by day 21 in treated CRC metastases.

Immunohistochemical staining for ACE localized this protein to the stromal desmoplasia surrounding angiogenic vessels in CRC metastases (Figure 3). In the normal liver adjacent to CRC metastases, ACE localized to the hepatic endothelial cells of liver veins and some sinusoidal endothelial cells in both sham operated animals and in tumor-bearing control and captopril treated animals. Because no obvious difference in the number of ACE-positive cells or in the localization of positively-stained cells were observed in the liver tissue, cell counts were not performed on the liver adjacent to CRC metastases. However, marked differences in the number of positively stained cells were observed in the stromal infiltrations within CRC metastases. At day 16 control tumors had significantly more ACE-positive cells compared to captopril treated CRC metastases, but ACE expression reverts back to the level seen in untreated tumors at day 21 (Figure 4). Thus, the ACE-positive cell counts reflected mRNA ACE expression in CRC metastases (Figure 2 and 4).

In contrast to ACE, ACE2 mRNA expression was lower in tumors compared to the surrounding liver at day 21 in control animals (*P *= 0.0011) (Table 2).

### Classical and alternative RAS receptors are differentially expressed in CRC liver metastases

AT1R expression was elevated by tumor induction at day 5 (*P *= 0.0030) (Table 2). AT1R expression in the liver was reduced by captopril treatment at days 5 (*P *= 0.0001) and 16 (*P *= 0.0003) (Figure 2). While in control animals CRC metastases had significantly lower expression than the surrounding liver (*P *= 0.019 day 16 and *P *= 0.0003 day 21), there was no difference between AT1R expression in CRC metastases and liver of captopril treated animals. Indeed, AT1R expression in the liver of captopril treated animals was significantly less than the expression detected in the livers of control animals (Table 2). In contrast to the AT1R, AT2R and MasR expression was higher in CRC metastases compared to the surrounding liver, reaching significance for the MasR (*P *≤ 0.0046) (Table 2). At day 16 MasR mRNA expression also increased in tumors with captopril treatment (*P *= 0.0299) compared to control CRC metastases (Figure 2).

**Figure 5:**
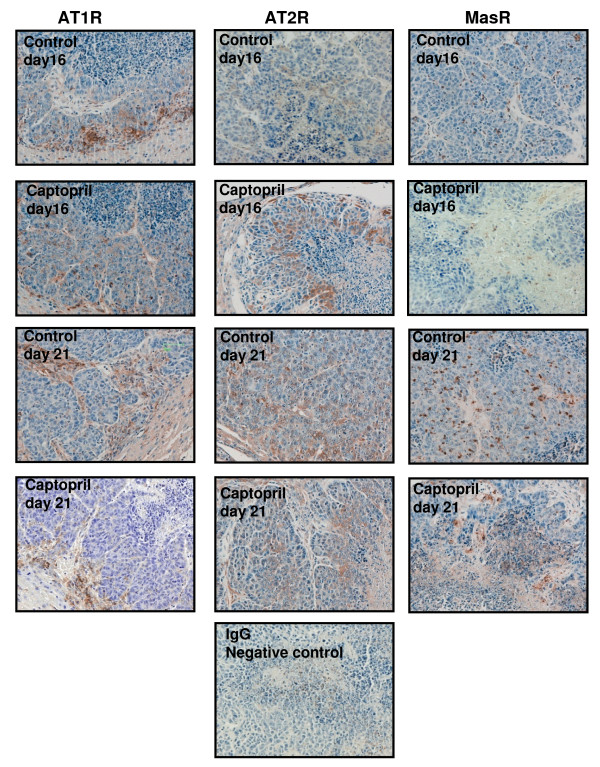
**Immunohistochemical staining for the AT1R (A), AT2R (B), and MasR (C) in control and captopril treated animals at day 16 and day 21**. AT1R localised to cells within the stromal intrusions of CRC metastases with light staining also observed in tumor cells at the periphery (A). There appeared to be a greater number of AT1R-positive cells in and around CRC metastases in control compared to the captopril treated animals, supporting mRNA analysis for this receptor (Figure 2). AT2R protein localized to tumor cells only with no staining evident in the liver (B). MasR staining was found within and around the necrotic centre of CRC metastases and a greater number of MasR staining was observed in control groups as compared to the captopril treated groups (similar to the mRNA data presented in Figure 2). As with the AT2R, MasR staining was only evident in tumor cells.

Immunohistochemistry for the AT1R showed localization to the stromal intrusions of CRC metastases in addition to cells lining the liver sinusoids (Figure 5). Staining on tumors cells was observed only at the periphery and was notably less intense than staining of the infiltrating stromal cells. Given their location surrounding CRC metastases and in the liver sinusoids it is likely that at least some of these AT1R-positive cells are tumor-associated macrophages (liver Kupffer cells). Consistent with our findings of AT1R mRNA expression, the number of these AT1R-positive cells appeared lower in captopril treated animals (see representative immunohistochemical images). While the AT1R localized to cells within the surrounding liver, with comparatively light staining on CRC metastases cells, only CRC metastases cells were found to stain positively for the AT2R and the MasR. These results support the finding that the mRNAs for both these alternative receptors was notably higher in CRC metastases than corresponding liver. The AT2R localized to cells near the proliferating rim (tumor-liver boundary), but, in contrast to the AT1R, did not extend to this edge. The MasR localized to cell in and around necrotic regions. The number of MasR-positive cells in control CRC metastases at day 16 and day 21 was noticeably higher and more consistent than captopril treated CRC metastases. Again these results support our mRNA analysis of this receptor.

## Discussion

RAS expression has been described in various cancer cells and tissues and there is strong evidence that ACE inhibition can inhibit tumor growth [7,9,11,32-37]. In our previous study, we found that the administration of the ACE inhibitor captopril caused a marked reduction in the volume of CRC liver metastases and this was confirmed in the present study which also demonstrated a local RAS in CRC liver metastases that is distinct from the hepatic RAS [8].

### The RAS of colorectal cancer liver metastases is distinct from the liver RAS

ACE, AT1R, MasR, and angiotensinogen were differentially expressed in control CRC liver metastases compared to the naïve (non-tumor bearing) and the tumor-bearing liver. In particular, CRC metastases were characterised by high ACE and MasR expression, but low AT1R and angiotensinogen mRNA levels compared to the liver. These results indicate a distinct CRC cell-associated RAS.

These cancer cells are derived from transformed epithelial cells of the colon and the difference in RAS expression of these cells compared to the tumor-bearing and naïve liver most likely represents a combination of differences due to transformation and tissue origin (colon versus liver). Hepatocytes are the major source of circulating angiotensinogen and so it is perhaps not surprising that CRC metastases have lower angiotensinogen levels [38]. These results also imply that much of the ANG II available to drive growth of CRC liver metastases is derived from local host production of angiotensinogen, which is then converted to ANG II via high ACE expression in tumors. The high levels of tumor-derived ACE described here support reports of an up-regulation of ACE mRNA in primary CRCs compared to corresponding nonlesional tissues [39]. However, our immunohistochemical analysis suggests that at least some of the expression of ACE in CRC metastases is derived from infiltrating cells. It is known that infiltrating macrophages, which typically associate with stromal intrusions, express ACE [40].

MasR expression was also markedly higher in CRC metastases compared to the surrounding liver. The MasR, as well as binding ANG-(1-7), was originally described as a protoncogene based on its ability to transform NIH 3T3 cells and its up-regulation in tumors may represent a normal oncogenic pathway for CRC development [41]. However, transgenic mice overexpressing ANG-(1-7) do not have increased tumor formation and MasR overexpression in the retina leads to increased cell death without tumorgenicity suggesting that the MasR itself is not oncogenic [42]. Importantly, increased MasR expression may provide a target for initiating anti-tumor responses since MasR activation by ANG-(1-7) mediates a number of potentially anti-angiogenic and anti-proliferative effects. Indeed, ANG-(1-7) has been found to reduce serum-stimulated growth in human lung cancer cell lines [28].

AT1R protein is commonly expressed in several cancers such as the bladder, pancreatic, ovarian and renal cancer pulmonary metastasis [7-19]. However, in our study we found that AT1R expression was lower in CRC metastases compared to the surrounding liver. AT1R protein localized mainly to the stromal intrusions in liver metastases with only light staining in the cancer cells themselves and confined to those cells at the proliferating border of tumors. Given their location within the tumor desmoplasia and liver sinusoids many of these AT1R-positive cells are likely to be Kupffer cells, resident liver macrophages. Macrophages may contribute to tumor growth by producing growth factors that enhance angiogenesis. These infiltrating AT1R-positive cells may have a role in mediating the effects of RAS blockade on tumor growth.

The results presented here support the hypothesis that malignancies maintain a local RAS that reflects the tissue in which the primary tumor developed. However, our results do not preclude interactions between tumor and host RASs and it is likely that both have paracrine effects on the other.

### Captopril treatment alters expression of the RAS in tumors

The significant reduction in ACE expression at day 16 following captopril treatment suggests that in addition to the inhibition of ACE activity, captopril also reduces ACE levels, presumably leading to an even greater inhibition of ANG II production. The timing of this reduction in ACE expression is of interest as it occurs during the critical exponential growth phase (day 10 to 19). However, in the late plateau stages (day 21 onwards) of CRC metastases growth, captopril treatment was associated with a reversion of ACE expression back to the levels seen in untreated tumors at day 21. In our previous study we stopped captopril treatment at day 21 and found survival was not significantly improved in treated animals compared to controls [8]. Our current results suggest a possible explanation for this, as the increased expression of ACE mRNA (and protein) at this time would require additional, or at least continued, captopril treatment to ensure sustained inhibition of ACE activity.

### Captopril altered the liver RAS

It is likely that much of the ANG II postulated to drive growth of CRC metastases in the liver is derived from angiotensinogen expressed by the liver parenchyma. We also show that under captopril treatment angiotensinogen expression in the host liver was significantly reduced. Thus, in the captopril treated liver, the production of ANG II would be severely compromised by the inhibition of its conversion from ANG I, the preferential production of ANG-(1-7), and the reduction of angiotensinogen expression, all of which would contribute to the reduced availability of ANG II to support tumor growth [43,44].

## Conclusions

In conclusion, the present study provides evidence of a complete local RAS in both the normal and tumor-bearing mouse liver. We show a marked up-regulation of ACE during CRC metastases development, which would presumably favor CRC metastases growth by increasing production of ANG II. Captopril treatment reduced CRC metastases ACE expression during the period of rapid growth, but ACE expression increased at late stages (day 21). This increase in ACE expression would need to be taken into consideration if targeting ACE in anti-cancer therapies. High MasR expression in CRC metastases, but not in the liver, suggests that infusion of ANG-(1-7) may also inhibit growth of CRC metastases. The data presented here indicates that the tumor RAS may be differentially regulated from the adjacent liver RAS. This independence may allow treatments to negatively target the tumor RAS, while allowing the liver to function and respond as normal.

## Competing interests

The authors declare that they have no competing interests.

## Authors' contributions

JN carried out qRT-PCR and immunohistochemistry and drafted the manuscript. EA carried out ACE positive cell counts, established IHC protocols, and revised the manuscript. PA participated in the design of the study and manuscript revision. JZ established ACE IHC protocols. CH participated in the design of probes and primers for qRT-PCR. CC conceived of the study and revised the final manuscript. All authors have read and approved the final manuscript.

## Pre-publication history

The pre-publication history for this paper can be accessed here:

http://www.biomedcentral.com/1471-2407/10/134/prepub
